# Urban wastewater analysis as an effective tool for monitoring illegal drugs, including new psychoactive substances, in the Eastern European region

**DOI:** 10.1038/s41598-020-61628-5

**Published:** 2020-03-17

**Authors:** Anna Maria Sulej-Suchomska, Agnieszka Klupczynska, Paweł Dereziński, Jan Matysiak, Piotr Przybyłowski, Zenon J. Kokot

**Affiliations:** 1 Gdynia Maritime University, Faculty of Entrepreneurship and Quality Science, Department of Commodity and Quality Science, 81-87, Morska Str., 81-225 Gdynia, Poland; 2 Poznan University of Medical Sciences, Faculty of Pharmacy, Department of Inorganic and Analytical Chemistry, 6, Grunwaldzka Str., 60-780 Poznań, Poland

**Keywords:** Environmental monitoring, Environmental social sciences

## Abstract

The use of illicit drugs causes unquestionable societal and economic damage. To implement actions aimed at combating drug abuse, it is necessary to assess illicit drug consumption patterns. The purpose of this paper was to develop, optimize, validate and apply a procedure for determining new psychoactive substances (NPSs) and classic drugs of abuse and their main metabolites in wastewater samples by using solid phase extraction (SPE) and high-performance liquid chromatography coupled with tandem mass spectrometry (HPLC-MS/MS). Moreover, detailed validation of the procedure was conducted. The developed SPE–HPLC-MS/MS procedure (within the sewage-based epidemiology strategy) allowed for the simultaneous, selective, very sensitive, accurate (recoveries ≥ 80.1%) and precise (CV ≤ 8.1%) determination of new and classic psychoactive substances in wastewater samples. This study is characterized by new scientific elements, especially in terms of the freeze-thaw and post-preparative stability of the selected psychoactive substances. This is the first time that NPSs (mephedrone and ketamine), the main metabolites of heroin (6-acetylmorphine, 6-AM) and marijuana (11-nor-9-carboxy-Δ9-tetrahydrocannabinol, THC-COOH) have been detected and monitored in Poland. This study is also the first to corroborate the data available from the EMCDDA and EUROPOL report and indicates that the retail market for cocaine is expanding in Eastern Europe.

## Introduction

Drug abuse and illicit drug trafficking is a global phenomenon that causes a broad spectrum of social, health and economic problems^1–4^. The European Monitoring Centre for Drugs and Drug Addiction (EMCDDA) and the United Nations Office On Drugs and Crime (UNODC) reported that drug-related problems are becoming increasingly complex, especially with regard to the extremely dynamic nature of the new psychoactive substances market, stimulants, misused medicines and problematic cannabis use^3–7^. Moreover, the verification of the presence of traditional illegal drugs (*i.e*., amphetamine, methamphetamine, ecstasy, etc.) in sewage samples is still needed because of environmental and forensic issues^8,9^. The environmental impact of synthetic drug production has been highlighted in the last EMCDDA and EUROPOL report^7^. Waste from drug production discharged into surface waters may harm aquatic life, can potentially contaminate the meat of cattle, which can affect the human food chain, and could further spread hazardous substances into the soil and waterways^7,10^. In this context, it is crucial to pay greater attention to developing new methodologies as tools for monitoring illicit drug consumption and its trends and drug trafficking to combat drug abuse and improve quality of life^11–17^.

Illegal drug use is mostly an unofficial activity. Consequently, traditional survey methods, such as general population interviews and surveys, can be inaccurate and may also produce results based on conjectures^2,8,18,19^. Conventional survey methods are not suitable for monitoring fast-changing drug markets over time^20^. Thus, an innovative approach (called sewage-based epidemiology, SBE) has been recently developed and enables the accurate estimation of drug concentrations based on the direct concentration measurements of a drug of interest (or its metabolites) excreted in urine in untreated wastewater samples^8,21–23^. Consequently, we and other research teams have developed and successfully applied this methodology to investigate drugs of abuse in wastewater samples collected in, *inter alia*, Italy^22,24–28^, Ireland^29^, Spain^30,31^, Poland^32^, Belgium^13^, Switzerland^26^, Norway^33^, the United Kingdom^26,34^, Croatia^23^, the USA^35^, China^20^ and Australia^36^. The SBE approach has been used to approximate local and national consumption, monitor the short- and long-term consumption trends over time, and identify the use of new designer drugs as well as changing habits and trends of their use^2,3^. The distribution of psychoactive substances and their main metabolites, such as ketamine (KET), mephedrone (MEPH), amphetamine (AMPH), methamphetamine (METH), 3,4-methylenedioxyamphetamine (MDA), 3,4-methylenedioxyethylamphetamine (MDEA), 3,4-methylenedioxymethamphetamine (MDMA, ecstasy), the main metabolites of cocaine (benzoylecgonine, BEC), heroin, and marijuana, on the drug market and of their final products in human excretions in the environment is a subject of global interest because it enables the estimation of drug consumption and ecological health^3,8^. The quantitative analysis of drugs of abuse in sewage samples for estimating illegal drug consumption is complementary to existing epidemiology-based methods and can ensure additional, evidence-based information^2,37–39^. Independent and timely data on the scale, type, and demographics of illegal drug use are essential for a better understanding of drug consumption patterns, as well as for developing improved procedures and operations that can ensure the maximum reduction of the adverse effects of drug abuse and illicit drug trafficking^2,40^.

Regardless of the researcher’s level of experience and the extent of investigations, the determination of illicit drugs in municipal wastewater is a great analytical challenge. The main problems include the lack of or very limited availability of standard reference materials, especially for NPSs; low or very low contents of many analytes in wastewater; the possible co-occurrence of components with similar physicochemical properties in one sample; and difficulties in standardizing the measurement results due to the varying size of sewage treatment plants and changing weather conditions^8,11,41^. Selecting a suitable drug biomarker is a crucial step that is necessary for appropriate detection, identification and quantification of target compounds. An analyte is suitable for use as a drug biomarker when it meets a number of requirements, i.e. it must be a specific compound originating solely from the drug, it must remain stable under the conditions encountered at a wastewater treatment plant (WWTP), it has to be excreted in urine and not extensively partitioned onto solids, and its concentration in excreted urine and wastewater must be adequately high^11^.

The main aim of the research presented in this paper was to develop, optimize, validate and apply an SPE-HPLC-MS/MS-based procedure for the determination of new psychoactive substances, such as ketamine and mephedrone, and classic illicit drugs, including amphetamine, methamphetamine, 3,4-methylenedioxyamphetamine, 3,4-methylenedioxyethylamphetamine, 3,4-methylenedioxymethamphetamine, and the main metabolites of cocaine (benzoylecgonine), heroin (6-acetylmorphine) and marijuana (11-nor-9-carboxy-Δ9-tetrahydrocannabinol) in wastewater samples. The developed procedure was used in a pilot study to estimate the occurrence of illicit drugs in sewage samples collected at a WWTP in Poznań (Poland). We evaluated the presence of ketamine and mephedrone in Poland for the first time, NPSs that have recently become drugs of choice in the illicit marketplace. KET and MEPH have been assigned priority among newly abused substances^7^. It is noteworthy that 6-AC and THC-COOH were also monitored and detected in wastewater in Poland for the very first time. This research is characterized by the inclusion of new scientific elements in terms of freeze-thaw and post-preparative stability of the investigated drugs of abuse. Moreover, comparisons of the determined levels of drugs of abuse and their metabolites in Poland (in the region of Eastern Europe) with their levels measured in other parts of Europe and the world were conducted. The performed study is the first to corroborate the data available from the EMCDDA and EUROPOL databases and indicates that the retail market for cocaine is expanding in Eastern Europe, and there is a large production scale of amphetamine in Poland.

## Materials and methods

### Chemicals and materials

All investigated compounds, such as ketamine, mephedrone, amphetamine, methamphetamine, 3,4-methylenedioxyamphetamine, 3,4-methylenedioxyethylamphetamine, 3,4-methylenedioxymethamphetamine, benzoylecgonine (BEC), 6-acetylmorphine (6-AM), 11-nor-9-carboxy-Δ9-tetrahydrocannabinol (THC-COOH) and their deuterated analogues used as internal standards (ISs), KET-D4, MEPH-D3, AMPH-D6, METH-D5, MDA-D5, MDEA-D5, MDMA-D5, BEC-D3, 6-AM-D3, and THC-COOH-D9, were obtained from Sigma-Aldrich (Seelze, Germany) as certified solutions. The analytical reference substances and internal standards were purchased at concentration levels of 1 mg/mL or 100 µL/mL in acetonitrile (ACN) or methanol (MeOH). Working solutions were prepared by diluting the stock solutions with MeOH and stored in the dark at −20 °C until analysis. MeOH and ACN (LC-grade), ammonium formate, and formic acid were purchased from Sigma-Aldrich (Seelze, Germany). Glass microfibre filters GF/A were obtained from Whatman (Kent, UK). All other reagents were obtained from J.T. Baker (Griesheim, Germany). Oasis MCX (60 mg, 3 mL) SPE cartridges were obtained from Waters (Milford, MA, USA), while a SPE-12G™ vacuum manifold from J.T. Baker (Griesheim, Germany) was applied for the loading of wastewater samples and for the drying of the cartridges. Deionized water was obtained from a Millipore Simplicity UV purification system (Waters, Milford, MA, USA).

### Sample collection

Pilot samples of wastewater were collected at a WWTP in Poznań in Poland. The WWTP serves approximately 1 200 000 population equivalents. The influent samples were collected by applying a time-proportional autosampler over a 24-h period from 7 a.m. to 7 p.m. All samples of untreated wastewater were collected into 3 L high-density polyethylene (HDPE) containers by using an autosampler programmed to sample 200 mL every 60 min. Sampling was performed during two periods: the autumn season (the 1st of October and the 19th of October in 2015) and the spring season (seven consecutive days between the 16th of March and the 22nd of March in 2016). No storm overflows occurred during the sampling period since no stormy weather or heavy rainfall was recorded. The wastewater samples, kept at low temperature, were usually transported to the laboratory within 0.5 h after collection. The unpreserved samples were processed immediately upon arrival at the laboratory.

### Sample preparation

**Figure 1:**
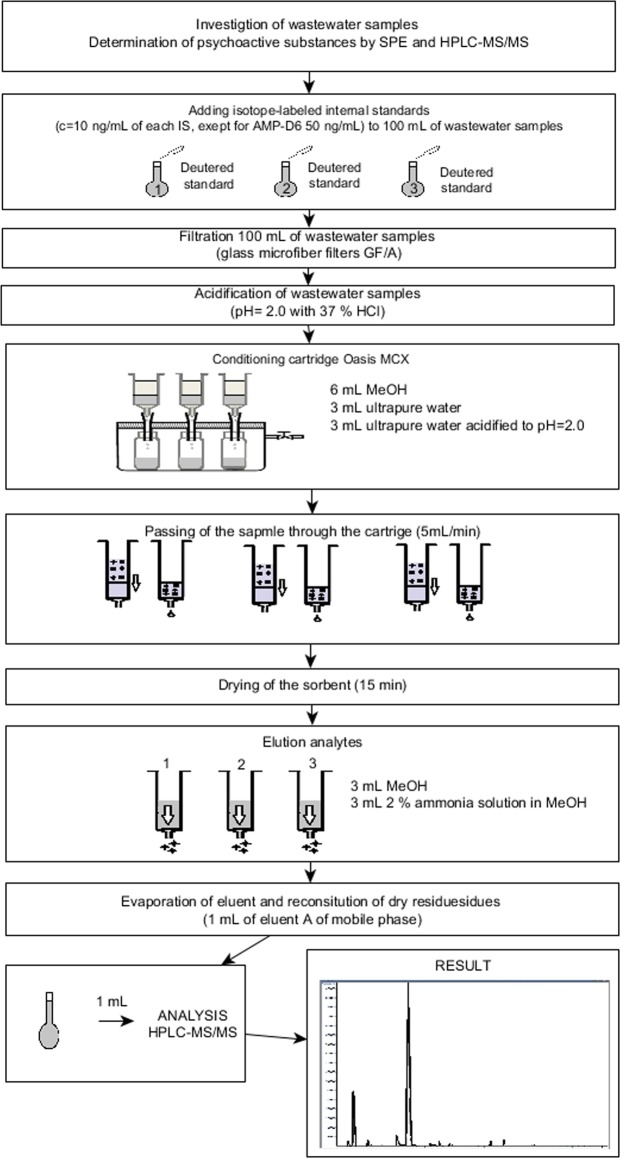
Schematic representation of the analytical procedure for the determination of illicit drugs in wastewater samples by using an SPE-HPLC-MS/MS-based system.

Sample preparation is a significant step in the analytical procedure used for the detection, identification and determination of psychoactive substances in samples with a very complex (and often variable) matrix composition^42^. Sample preparation and analysis were performed according to the procedure proposed by Castiglioni *et al*., which has been successfully used by many research teams for the determination of illicit drugs in environmental samples^27,32,43^. The appropriate amounts of a mixture of isotope-labelled illicit drug standards were added to the samples^32,44^. A schematic representation of the analytical procedures used for the determination of illicit drugs in wastewater samples is shown in Fig. 1.

### High-performance liquid chromatography–tandem mass spectrometry

Analyte separation was achieved by using a 1260 Infinity high-performance liquid chromatography (HPLC) system (Agilent Technologies, Santa Clara, CA, USA). The separation of target compounds was performed on an XTerra C18 column (100 mm × 2.1 mm, 3.5 mm, Waters, Milford, MA, USA) maintained at 35 °C. The mobile phase was composed of (A) 5 mM ammonium formate solution with 0.1% formic acid and (B) ACN with 0.1% formic acid and 10% solvent A. The following gradient elution was utilized: 0–5 min, 2% B; 5–14 min, linear increase from 2% to 90% B; 14–16 min, 90% B; 16–18 min, linear increase from 90% to 2% B; and 18–25 min, 2% B. The flow rate was set at 0.3 mL/min during the whole process, and the injection volume was 20 µL.

A 4000 QTRAP triple quadrupole mass spectrometer (Sciex, Framingham, MA, USA) equipped with an electrospray ion source (ESI) was used for the identification and quantification of illegal drugs.

Quantitative mass spectrometry analysis was performed in multiple reaction monitoring (MRM) mode by analysing the fragmentation products of the protonated or deprotonated pseudo-molecular ions of each drug and each deuterated analogue (Table 1). All analytes were determined in positive ionization mode, except for THC-COOH and THC-COOH-D9, for which negative ion mode was applied. THC-COOH demonstrated more abundant ionization in negative ion mode due to the relatively strong ionization affinity of the acidic group^30^. The acquisition was divided into two time segments to allow for analysis in different ionization modes^20^. For all analysed drugs of abuse and their deuterated analogues, two MRM ion transitions were monitored^45^. Several collision energies were tested to obtain the optimum response for each transition. The most intense transition was selected for quantification, while the second most intense transition was selected for confirmation of the analyte identity. Confirmation of positive findings was conducted by calculating the peak area ratios between the quantification (MRM_1_) and confirmation (MRM_2_) transitions. Next, the ratios were compared with the mean MRM_1_/MRM_2_ values obtained from the calibration standards for each analyte. The detection was considered positive when the obtained ion ratio fell within the tolerance range, in accordance with the EU guidelines for LC-MS/MS analysis (Commission Decision 2002/657/EC)^45^. Retention time was the other crucial criterion for compound identification, where the tolerance range was within ±2.5% of the retention time of the reference standard. The Turbo V™ source (Sciex, Framingham, MA, USA) settings were optimized using flow injection analysis (FIA) under the following conditions: ion spray voltage (ISV), 4500 V; source temperature, 500 °C; curtain gas (CUR), 30 psig; collision gas (CAD), medium; ion source gas 1 (GS1), 70 psig; and ion source gas 2 (GS2), 60 psig. Nitrogen was used as the curtain and collision gas. The optimization of MS parameters was performed individually for each drug in continuous-flow mode through direct infusion of standard solutions (0.01 μg/mL) in methanol/water (50:50, v/v). The optimized MS compound-dependent parameters, such as the declustering potential (DP), collision energy (CE) and cell exit potential (CXP) voltages for target analytes, are shown in Table 1. The abovementioned parameters were optimized by infusion of a single standard solution at a flow rate of 10 μL/min using a syringe pump (11 Plus, Harvard Apparatus, Holliston, MA, USA) that was directly connected to the interface. Dwell times of 22 and 69 ms per ion pair were used to determine analytes in positive ionization mode and negative ionization mode, respectively.

**Table 1:** Retention times (*t*_*R*_) and optimized MRM conditions used for HPLC-MS/MS analysis of psychoactive substances in wastewater samples.

Compound	Abbreviation	t_R_ (min)	Molecular mass (Da)	DP (V)	MRM_1_^a^ (Quantifier)	CE 1 (V)	CXP 1 (V)	MRM_2_^b^ (Qualifier)	CE 2 (V)	CXP 2 (V)	MRM ratio^c^ (MRM_1_/MRM_2_)
6-acetylmorphine	6-AM	10.80	327.4	110	328.2 → 165.1	55	30	328.2 → 211.1	38	12	1.1
6-acetylmorphine -D3	6-AM-D3	10.80	330.4	71	331.2 → 211.2	37	40	331.2 → 271.2	37	45	3.1
Amphetamine	AMPH	9.10	135.2	30	136.1 → 91.1	26	16	136.1 → 119.1	9.0	11	3.9
Amphetamine-D6	AMPH-D6	8.85	141.2	51	142.1 → 93.1	23	16	142.1 → 125.1	14	12	1.5
Benzoylecgonine	BEC	11.50	289.3	75	290.1 → 168.1	29	16	290.1 → 105.1	45	19	2.3
Benzoylecgonine-D3	BEC-D3	11.50	292.3	81	293.1 → 171.1	30	16	293.1 → 105.1	53	10	8.3
Ketamine	KET	11.30	237.7	64	238.1 → 125.1	49	21	238.1 → 179.2	26	16	2.6
Ketamine-D4	KET-D4	11.30	241.7	61	242.1 → 211.2	24	11	242.1 → 224.1	25	21	1.0
Mephedrone	MEPH	11.00	177.2	61	178.2 → 145.1	30	25	178.2 → 144.1	39	23	2.0
Mephedrone- D3	MEPH-D3	11.00	180.2	61	181.2 → 148.2	30	25	181.2 → 163.2	20	16	1.9
Methamphetamine	METH	10.40	149.2	31	150.1 → 91.1	29	16	150.1 → 119.1	17	10	2.6
Methamphetamine-D5	METH-D5	10.40	154.2	50	155.1 → 121.1	16	16	155.1 → 92.1	28	12	1.0
3.4-methylenedioxyamphetamine	MDA	10.50	179.2	28	180.2 → 105.1	32	18	180.2 → 135.1	27	12	1.7
3.4-methylenedioxyamphetamine-D5	MDA-D5	10.50	184.3	51	185.2 → 168.1	17	16	185.2 → 110.1	33	10	4.2
3.4-methylenedioxy-methamphetamine	MDMA	10.80	193.2	55	194.1 → 163.1	18	14	194.1 → 105.1	37	9.0	2.4
3.4-methylenedioxy-methamphetamine-D5	MDMA-D5	10.80	198.2	70	199.1 → 165.1	19	14	199.1 → 107.1	34	8.0	3.1
3.4-methylenedioxy-ethylamphetamine	MDEA	11.20	207.3	45	208.1 → 163.1	20	14	208.1 → 105.1	37	8.0	2.4
3.4-methylenedioxy-ethylamphetamine-D5	MDEA-D5	11.20	212.2	52	213.1 → 163.1	20	8.0	213.1 → 105.1	37	9.0	2.8
11-nor-9-carboxy-Δ9-tetrahydrocannabinol	THC-COOH	16.90	344.5	−105	343.0 → 299.1	−30	−12	343.0 → 325.1	−28	−15	7.7
11-nor-9-carboxy-Δ9-tetrahydrocannabinol-D9	THC-COOH- D9	16.90	353.5	−117	352.0 → 308.1	−30	−12	352.0 → 334.2	−30	−17	2.0

^a^Precursor ion → product ion I ^b^precursor ion → product ion II; DP: declustering potential; EP: entrance potential; CE: collision energy; CXP: collision cell exit potential; MRM: multiple reaction monitoring.

Instrument control, data acquisition, processing and evaluation were performed with Analyst software version 1.5.2 (Sciex, Framingham, MA, USA).

### Quality assurance/quality control (QA/QC)

A validation process was conducted to determine the selectivity, linearity, range, trueness, method detection limits (MDLs), method quantification limits (MQLs), intra-day (within-run) precision and inter-day (between-run) precision of the procedure.

Quantitative analysis based on peak areas was carried out by using the internal standard method. For each studied compound, the corresponding deuterated analogue was used as the IS. The concentrations of analytes were calculated by using the standard calibration curves, which had been generated using the detector response. The analyte concentrations were expressed as the ratio of the analyte ion (the specific, most abundant product ion) to the base peak ion of the internal standard.

Procedure selectivity was verified by examining the chromatograms for the presence of any interfering peaks at the target analyte retention time for each MRM transition. No relevant interfering peaks were detected at the retention times of the target analytes. In addition, the use of MRM mode, with the calculation of the MRM ratio, provided sufficient selectivity of the proposed methodology. The procedure was determined to be highly specific.

The linearity and range of the developed methodology were determined by serial dilution of a stock solution of the investigated psychoactive substances (1 mg/mL). An eight-point calibration curve, in the range of 1.0–1000 ng/L, was constructed by injecting mixed standard solutions containing different amounts of each compound and a fixed amount of each IS (20 ng/L, except for AMPH-D6, which was at 100 ng/L). The first point of the calibration curve was always that of an instrumental blank sample. The curves were freshly prepared prior to each sample analysis by diluting the stock solution. Each calibration point was analysed in triplicate.

The limits of detection (LODs) and limits of quantitation (LOQs) were determined by using the signal-to-noise approach. The LODs and LOQs for the target analytes were defined as analyte concentrations giving signal-to-noise ratios (S/N) of 3 and 10, respectively. The limits of detection (LODs) were determined based on triplicate measurements. These results were used to establish the method detection limits (MDLs) and method quantification limits (MQLs) of the analytical methodology, taking into account all analytical steps from sampling to statistical data analysis. The MDL was defined as the lowest concentration of an analyte that could be detected with a specified probability using a given analytical procedure. In turn, the MQL was the lowest concentration of an analyte that could be quantified with a certain precision, accuracy and uncertainty using the proposed analytical procedure.

Trueness studies were carried out by analysing wastewater samples containing low, medium and high concentration levels of the target analytes. Blank wastewater samples (100 mL) were spiked with 10, 100 and 500 ng/L of each investigated compound. Moreover, the wastewater samples were spiked with 100 ng/L of each isotopically labelled analyte (500 ng/L AMPH-D6) prior to every filtration and extraction process. The concentration levels of spiked samples were estimated based on the expected analyte concentrations in real samples. It was complicated to obtain genuine blank samples of wastewater. Blank samples were collected from the WWTP during the weekdays when lower concentration levels of illicit drugs were expected than on the weekends. These blank samples were analysed before each analysis. The recovery of the analytical procedure was tested by analysing wastewater samples in triplicate at each spiked concentration. As mentioned before, the recovery values were assessed by the standard addition method. Known amounts of analyte(s) and isotopically labelled standards were added to one sample, while parallel samples were only spiked with isotopically labelled standards. After completing the analysis of the sample without the added analytes (x) and the sample containing the added analytes (x + s_i_), the recovery was estimated using the equation of % recovery = [(x + si) − (x)/s] × 100, where s_i_ is the amount of the determined analyte^46^.

The precision of the methodogy was evaluated by spiking wastewater samples with the target analytes and labelled standards, as previously described in the section on trueness. The precision was expressed as the coefficient of variation (CV) of replicate measurements according to the following equation: CV = SD/*X* × 100%, where SD is the standard deviation of the concentration of the target analyte and *X* is the average concentration of the target analyte in a sample. The determination of precision included both inter-day and intra-day assays. The intra-day assay included three independent runs of each QC sample at three concentration levels in a single batch for one day. The inter-day precision was estimated based on the analysis of QC samples (three replicates per sample) at three concentration levels on separate days within a three-day period.

## Results and discussion

In this study, we used the latest National Report and the EMCDDA Report to identify illegal drugs that pose a substantial hazard to public safety^3,7,47^. Other aspects of the selection process included the following criteria: stability of the psychoactive substances or their active metabolites in wastewater under the conditions encountered at a WWTP; specificity of the biomarker that originates exclusively from the drug of abuse; and relatively high concentration levels of the target compounds in excreted urine and wastewater. The availability of proper internal standard solutions for selected compounds was also taken into account. Considering the aforementioned issues, the wastewater samples were analysed for the presence of 10 drugs of abuse and their metabolites by using a fully validated analytical procedure based on SPE-HPLC-MS/MS.

### High-performance liquid chromatography–tandem mass spectrometry

Chromatographic separation is not a decisive issue when using MS/MS for detection since the probability of finding two compounds with identical retention times and the same MRM transitions is quite low. However, in the case of HPLC-MS/MS methods, efficient HPLC separation is crucial to avoid or eliminate matrix effects. Furthermore, the selection of the mobile phase composition might be important because it can enhance the detector response^8,30^.

In this work, a C_18_ column was used to separate target compounds. Based on data in the literature and the authors’ prior experience, it was concluded that this type of column would provide the best separation of the investigated compounds^27,32,48,49^. To obtain the optimal chromatographic separation and ESI ionization, different mobile phases were tested, which differed with respect to their content of organic solvent, pH, concentration, and the type of buffer. Additives, such as ammonium acetate, ammonia, ammonium formate or alkylamines, are known for suppressing the signal in ESI+. Acidic additives support protonation of basic molecules, which, consequently, produces stronger signals when the ESI source is operated in positive mode. On the other hand, acetic and formic acids added to the mobile phase at various concentrations have been found to provide good separation and sensitivity with ESI source^50^. Formic acid at a concentration of 0.1% was selected as a mobile phase additive for this procedure. The chosen mobile phase and the type and temperature of the column (conditions identical to those proposed by Castiglioni *et al*.^27^) provided the highest peak areas and the best peak shape and resolution of the analytes. The HPLC mobile phase was directly introduced into the ion source without splitting. The total analysis time was 25 min, but the periods of 0–3 min and 20–25 min were sent to waste to protect the analytical column and to avoid contamination of the ion source with matrix components. The described method resulted in short retention times (8.5 to 17 min) for all investigated compounds. Consequently, a relatively fast and advantageous method was developed.

Optimal values of the MS parameters for each analyte and internal standard compound are shown in Table 1. Examples of extracted ion chromatograms of a solution of the investigated drugs of abuse at a concentration of 0.01 µg/mL in methanol/water (50:50, v/v) in MRM mode are shown in the SI, Fig. S1. The optimized procedure allowed for the successful separation of the investigated psychoactive substances in less than 17 min.

### Solid phase extraction

An important aspect of multi-residue analysis of drugs of abuse is the selection of the best SPE absorbent that allows for an acceptable level of analyte recovery. In this study, the extraction process was carried out with Oasis MCX cartridges, which are mixed reversed phase/cation exchange cartridges^27^. The MCX sorbent allows for both reversed phase and ion-exchange interactions. The mixed mode polymeric sorbent ensures sufficient selectivity towards the investigated compounds due to pH and polarity changes during the loading, washing and elution steps^40^.

The target analytes were analysed by injecting the extracts obtained after SPE and quantified by using calibration curves constructed by using standards dissolved in mobile phase A. Several strategies are commonly applied to handle matrix effects in quantitative analysis, but the most satisfactory approach is based on the use of stable isotope-labelled internal standards. Therefore, isotopically labelled standards were added to the investigated samples as surrogate internal standards. The extraction efficiency of each target compound was estimated from the corresponding recovery percentage. Relative recoveries (relative to the recovery of the surrogate/internal standard) obtained for the investigated drugs of abuse in sewage samples are presented in Table 2. All analytes showed satisfactory recovery values (80.1–119.4%), which were similar to those reported in the literature^23,40,49^.

**Table 2:** Performance data for the analysis of drugs of abuse (relative recovery in wastewater matrices, intra-day precision, and inter-day precision).

Compounds	R^2a^	Linearity range	MDL	MQL	Recovery (n = 3)									Precision, CV(n = 3)											
		[ng/L]												Intra-day CV [%]			Inter-day CV								
					[%]									[%]											
					Low			Medium			High			Low	Medium	High	Low			Medium			High		
					D1^b^	D2^c^	D3^d^	D1	D2	D3	D1	D2	D3				D1	D2	D3	D1	D2	D3	D1	D2	D3
6-AM	0.9901	1.0–1000	0.5	1.0	88.5	80.4	80.1	98.6	97.5	100.8	99.6	101.3	96.6	4.6	4.1	5.9	7.2	4.3	2.3	2.8	4.6	4.6	4.3	2.3	5.3
AMPH	0.9954	5.0–1000	1.0	5.0	98.7	100.0	104.4	112.2	93.4	106.4	103.7	99.8	93.8	3.8	3.5	3.5	2.4	5.6	3.4	4.6	2.6	3.3	2.7	5.3	2.6
BEC	0.9935	1.0–1000	0.1	0.5	98.3	88.9	102.8	106.2	101.8	100.6	98.9	99.1	102.0	3.1	2.8	4.4	2.9	3.9	2.6	3.7	3.3	1.4	3.7	4.9	4.5
KET	0.987	1.0–1000	0.1	0.3	91.8	94.7	99.3	95.6	101.9	99.4	101.0	100.5	102.6	3.8	4.2	4.2	2.6	4.8	3.9	4.5	4.3	5.3	4.9	4.3	3.4
MEPH	0.9916	5.0–1000	0.1	0.3	111.6	97.3	88.1	108.8	106.1	96.2	100.3	97.5	99.6	5.9	5.2	3.5	8.0	4.7	5.0	7.4	2.3	5.8	2.7	3.5	4.2
METH	0.9954	1.0–1000	0.1	1.0	98.8	93.8	103.1	115.6	118.9	115.3	101.2	105.8	102.0	5.2	5.6	5.4	7.2	5.4	3.1	6.2	4.8	5.9	4.7	5.9	5.6
MDA	0.9939	5.0–1000	2.0	5.0	103.4	98.6	105.0	119.2	117.6	118.9	118.9	119.4	114.9	6.3	2.6	2.9	8.1	7.1	3.7	1.8	1.1	4.8	3.6	1.6	3.5
MDMA	0.9944	1.0–1000	0.1	0.3	100.5	98.3	104.4	103.6	88.2	110.6	101.6	117.5	108.8	2.4	3.6	3.6	0.9	1.5	4.8	2.1	3.3	6.4	4.5	5.5	7.4
MDEA	0.9944	1.0–1000	0.3	0.5	112.5	112.9	113.2	113.3	117.1	117.0	116.4	117.1	111.0	5.5	2.3	2.1	5.6	3.2	7.8	2.5	2.1	2.4	2.6	1.5	2.2
THC-COOH	0.988	5.0–1000	1.0	3.0	96.7	103.9	115.6	86.1	95.9	112.7	103.9	98.7	82.4	3.4	4.4	4.4	4.5	2.3	3.3	2.4	4.9	8.1	3.1	4.9	5.1

^a^R^2**-**^Coefficient of determination, ^b^D1-day 1, ^c^D2-day 2, ^d^D2-day 3.

### Quality assurance/quality control (QA/QC)

After the optimization process, the developed methodology for determining illegal drugs of abuse in wastewater samples was validated to ensure the appropriate level of quality control and assurance of the results. The values of the validation parameters for the developed procedure are presented in Table 2.

The mean coefficients of determination (R^2^) of the calibration curves were higher than 0.990, which indicated good linearity. Only in the case of the calibration curves for THC-COOH and KET did the coefficients of determination equal 0.988 and 0.987, respectively. The calibration curve ranges were slightly different depending on the analyte (Table 2). Moreover, the concentrations of the investigated drugs of abuse and their metabolites in wastewater samples fell within the tested linearity range.

The proposed procedure was validated for 10 analytes, with MDL and MQL values ranging from 0.1 to 2.0 ng/L and from 0.3 to 5.0 ng/L, respectively. The sensitivity of the procedure was similar to that reported by Baker *et al*.^51^, Gonzales-Martino *et al*.^52^, Senta *et al*.^53^, and Mackuľak *et al*.^54^. It can be stated that the obtained MQL values were sufficiently low for the determination of the investigated drugs of abuse in wastewater samples. Some differences in the method detection limits and method quantification limits for the target analytes were observed. The noticeable differences in sensitivity may be connected to the differences in precursor ion formation and transmission as well as the variation in the fragmentation behaviour of the analytes.

The obtained recoveries were sufficient for all studied compounds (≥80.1%) at every fortification level (Table 2). These values also met the requirements of analytical methods, where the recovery should range from 70 to 120% depending on matrix complexity^46^. The performed studies showed that the efficiency of the developed analytical methodology was similar to that reported by González-Mariño *et al*. (95–116%)^52^, Du *et al*. (81.8–86.7%)^49^, Daglioglu *et al*. (85–114%)^16^ and Baz-Lomba *et al*. (87.8 and 113.1%)^55^.

The values of intra-day precision for the samples of wastewater ranged from 2.1 to 6.3%, while the values of inter-day repeatability ranged between 0.9 and 8.1%. The precision data for all target compounds are listed in Table 2. The precision studies demonstrated that the repeatability of the proposed procedure was similar to that achieved with other procedures^52,56^ and slightly better than that achieved by Celema *et al*.^57^ and van Nuijs^58^.

### Stability in wastewater

The stability of drugs of abuse in a complex matrix, such as wastewater, is a function of storage conditions, the physicochemical properties of the target compounds, biochemical changes, the matrix itself, and the containers utilized. If we anticipate the use of collected samples over a longer period of time, we should establish their stability^59,60^.

The stability of each psychoactive substance in the investigated samples was studied by determining short-term stability, freeze-thaw stability and post-preparative stability. The freeze-thaw stability test determined the degree of analyte loss after a number of freeze-thaw cycles. The applied test consisted of three repeated cycles, i.e., sample freezing at −20 °C for 24 h followed by unassisted sample thawing at room temperature^59^. The short-term stability test allowed for the assessment of changes in the concentration levels of analytes in unprepared samples stored for a few or several hours at room temperature. Thus, the collected samples were first frozen and thawed at room temperature and then kept at room temperature for 4 h prior to HPLC-MS/MS analysis. On the other hand, post-preparative stability testing allowed for the assessment of samples after the extraction, which were stored in the autosampler for several hours prior to analysis. In this case, the stability of the processed samples was determined by analysing the extracts of wastewater samples 8 h after extraction^59,60^.

The results of the stability tests were expressed as percentage differences between the initial and final concentrations of the target compounds. Each stability assay was conducted by analysing the samples in triplicate, and the concentration level of each analyte was 10 ng/mL. All stability determinations were carried out by using samples prepared from a freshly made stock solution of the analyte. The results of the performed stability tests are presented in Table 3. The target compounds in prepared wastewater samples were defined as stable if the mean concentration in the stored samples fell within ±15% of the initial concentration^59^. The values of freeze-thaw stability for the analytes in wastewater samples ranged from 2.8 to 9.7%. Only in the case of mephedrone and ketamine were the freeze-thaw stability values 26.6% and 18.9%, respectively. The test results of short-term stability and post-preparative stability ranged from −6.0 to 14.6% and from −7.2 to 3.5%, respectively. In the case of the short-term stability of the target analytes, relatively similar results have been reported in the available literature data^61,62^. Considering the aforementioned results, it can be concluded that all studied compounds fell within an acceptable range, except for ketamine and mephedrone, which showed poor stability during freeze-thaw stability testing.

**Table 3:** Stability tests performed for the investigated drugs of abuse in wastewater samples.

Compounds	Freeze-thaw stability		Short-term stability		Post-preparative stability	
	difference [%]^a^	SD [%]	difference [%]	SD [%]	difference [%]	SD [%]
MDA	0.9	0.3	−0.3	2.4	2.2	2.2
AMPH	2.6	1.2	−4.6	1.5	1.1	1.3
MET	3.8	1.1	−6.0	1.5	2.2	2.5
MDEA	9.7	0.6	0.1	2.4	3.5	0.1
MDMA	8.0	2.0	4.3	0.7	−0.5	0.5
BEC	2.8	0.8	0.2	0.1	2.2	1.6
MEPH	26.6	3.6	14.6	1.8	−4.5	1.9
KET	18.9	2.1	11.9	1.1	1.6	0.8
6-AM	6.8	3.2	1.8	4.9	−5.0	0.7
THC-COOH	6.3	9.3	5.6	0.8	−7.2	0.5

^a^Difference between the initial and final concentrations of target compounds.

Literature data on stability studies of the investigated drugs of abuse in wastewater are limited^7,11,13,14,53,61–66^. There are clear gaps in the results of stability tests of various drugs of abuse in wastewater. This study is characterized by new scientific elements, especially in terms of the freeze-thaw and post-preparative stability of KET, MEPH, BEC, THC-COOH, MDA, MDEA, AMPH, 6-AC, METH, and MDMA. The stability of drugs of abuse in wastewater is a very important issue, especially in the case of freeze-thaw stability. There are numerous situations that may require repeated freezing and thawing of samples. For example, samples can be prepared in the laboratory close to the sampling site (i.e., wastewater treatment plant) and then frozen prior to their transport and subsequent analysis in a laboratory equipped in an LC-MS system. Moreover, the prepared samples may be sent to another control/reference laboratory, or there may be a need to reanalyse some samples later on. Under all circumstances, repeated freezing and thawing of samples is required. Thus, it is crucial to test the short- and long-term stability of samples, including freeze-thaw cycles, to ensure good quality of the results. The results of the stability studies complement the database of the stability of illicit drugs in wastewater. However, this research topic is not yet fully understood and requires further research.

### Environmental application

**Figure 2:**
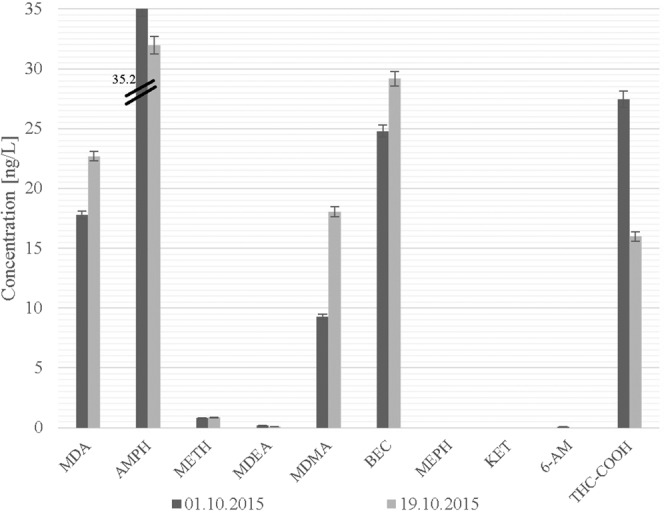
The concentrations of drugs of abuse in wastewater samples collected at a WWTP in Poznań (Poland) in autumn 2015.

**Figure 3:**
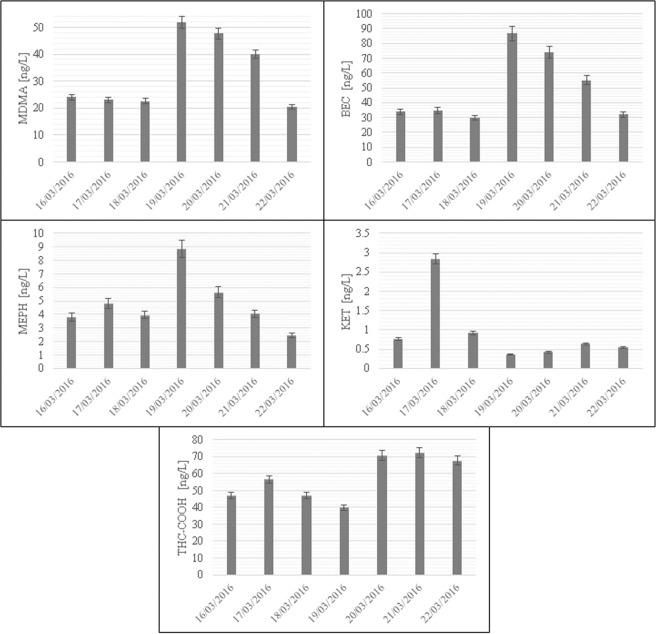
The concentrations of illicit drugs in wastewater samples collected at a WWTP in Poznań (Poland) in spring 2016.

The validated SPE-HPLC-MS/MS-based procedure was applied to determine the contents of new psychoactive substances and classic drugs of abuse and their metabolites in wastewater samples collected at a WWTP in Poznań, Poland. Samples of untreated wastewater (influents) were collected from autumn 2015 to spring 2016. In Fig. S2, the chromatograms (in both positive and negative ion modes) of drugs of abuse extracted from a wastewater sample collected at the WWTP in Poznań (Poland) on the 21^st^ of March 2016 are presented. The highest peak in the analysed chromatograms was attributable to amphetamine. It should be noted that the investigated drugs of abuse were sufficiently separated from interfering compounds present in the wastewater. The concentration levels of individual drugs of abuse determined in wastewater samples collected at the WWTP during two sampling campaigns are presented in Figs. 2 and 3. Amphetamine and the main metabolites of both cocaine (BEC) and marijuana (THC-COOH) were most abundant during the entire sampling period, with in the range of 31.9–139.9, 24.8–86.7, and 16.0–72.4 ng/L, respectively. Additionally, high concentrations of ecstasy (9.3–51.8 ng/L) were determined in all samples collected during this period. On the other hand, the new psychoactive substances ketamine and mephedrone were only determined in samples collected in spring 2016 (concentrations in the range of 0.4–2.8 and 2.4–8.9 ng/L, respectively). The determined contents of ketamine and mephedrone in wastewater samples confirmed the presence of these new psychoactive substances on the drug market in Poland. In the case of the heroin metabolite 6-AM, its low concentrations (0.1 ± 0.02 ng/L) were only measured in wastewater collected in autumn 2015 (1 Oct 2015). In general, the contents of the target drugs of abuse in samples collected during the spring sampling campaign were substantially higher than those determined in samples collected during the autumn sampling campaign (see Figs. 2 and 3). For example, the concentrations of AMPH, MDMA and THC-COOH in wastewater samples collected during the spring sampling campaign were ca. 3.0, 2.4 and 2.7 times higher than in the wastewater samples collected during the autumn sampling campaign. Exceptions to this trend were the concentrations of MDA and MDEA in samples collected in autumn 2015, which were in the range of 17.8–22.7 and 0.1–0.21 ng/L, respectively. During the spring sampling campaign, the concentration levels of these two drugs of abuse in wastewater fell under the MDLs. Considering the seven days of the spring sampling campaign, it was noted that the concentrations of amphetamine, ecstasy, and cocaine metabolites in the samples collected during the weekend were substantially higher than those measured in samples collected on weekdays.

When considering a broader context of the high concentration level of the main metabolite of cocaine (BEC) determined in all investigated wastewater samples, it can be concluded that the obtained results are the first to confirm the data included in the latest EMCDDA and EUROPOL report, which indicates that the retail market for cocaine is expanding in Eastern Europe^7^. Moreover, according to the abovementioned report, Poland ranks ninth in the retail market for cocaine in Europe and may therefore be viewed as the largest cocaine market in Eastern Europe. However, the level of benzoylecgonine measured in the wastewater collected from Poland was still low compared to that detected in wastewater from the western and southern regions of Europe^52,67^ and dramatically low compared to the levels of BEC determined in sewage samples collected from North America (241–1247 ng/L)^68^. The concentration level of the main metabolite of cocaine determined in wastewater from Poland was comparable to that in countries in the northern region of Europe^33^.

During our study, the highest concentration level of the investigated drugs of abuse was measured for amphetamine. The source of this compound is from both drug users and illegal drug producers. In the analyses of amphetamine from wastewater, a part of the drug concentration in the sewage system may also come from methamphetamine users because amphetamine is a metabolite of methamphetamine. The conducted research is the first to corroborate the data available from the latest EMCDDA and EUROPOL report, which summarizes that Poland is the third highest amphetamine-producing country in Europe, with 32 amphetamine production sites between 2015 and 2017^7^. The concentration levels of amphetamine determined in wastewater reported for other southern European counties, such as Croatia (14.0–545.0 ng/L) and Spain (194.0–7565.0 ng/L), were much higher than that in the present study, which occurred in the region of Eastern Europe. The level of amphetamine residues determined in this study was similar to those reported in sewage samples collected from the Czech Republic (23.0–80.0 ng/L) and much higher than that determined in samples collected from a WWTP in Canada (23.0–43.0 ng/L).

The methamphetamine concentrations in wastewater samples ranged from 1.2 to 1.4 ng/L. The concentration level of this analyte was very low when we take into consideration widespread use of this drug in neighbouring countries, e.g., the Czech Republic (13.0–1584 ng/L) and Slovakia (244.0–759.0 ng/L). For comparison, a very high concentration of METH was also determined in wastewater samples collected in China (244.0–759.0 ng/L)^54^.

Another aspect that was first compared with the data from the EMCDDA and Europol report was the confirmed presence of NPSs in wastewater from Poland, where, in 2016, two production sites of mephedrone were reported. Generally, the ketamine content in samples collected from the Polish WWTP was lower (<MQL-2.8 ng/L) than those determined in samples from WWTPs in the western (Netherlands, 10.0–17.0 ng/L; Belgium, 5.0–29.0 ng/L) and northern regions of Europe (United Kingdom: 52.0 ng/L)^13,20,69^. Regarding the available data, the highest concentration of KET was measured in samples collected from a WWTP in China (5–500 ng/L)^20^. Moreover, the contents of mephedrone determined in wastewater from Poland were higher (2.4–8.9 ng/L) than those determined in the samples from western and southern Europe (Belgium, Spain: <MQL)^13,52^.

In this study, a relatively high concentration of the main marijuana metabolite was also noted. Marijuana is a soft drug that is relatively inexpensive, easily accessible or even self-produced. The monitored samples come from the area of a large student city with an increased concentration of young people. The high concentration of THC-COOH measured in the monitored samples could be related to the popularity of cannabis among young people in Poland.

## Conclusion

As a result of this study, a reliable SPE-HPLC-MS/MS-based methodology for the simultaneous determination of new psychoactive substances and classic drugs of abuse and their metabolites in wastewater samples was developed, optimized and validated. It should be emphasized that a very detailed validation process was conducted in this study. The established procedure is selective, very sensitive, accurate, precise, inexpensive, relatively rapid and easily applicable to the analysis of sewage samples. Our study is characterized by new scientific elements in terms of freeze-thaw and post-preparative stability of the investigated drugs of abuse and their metabolites. The established analytical procedure was successfully applied to determine illegal drugs of abuse in real samples collected at a wastewater treatment plant located in the Eastern European region (Poland). This is the first time that mephedrone, ketamine, the main metabolites of heroin (6-acetylmorphine) and marijuana (11-nor-9-carboxy-Δ9-tetrahydrocannabinol) have been detected and monitored in Poland. The research results confirmed the presence of both new designer drugs and classic drugs of abuse on the Polish drug market. The conducted research is unique when we take into account the monitoring of wastewater in the region of Eastern Europe. Only one other research team has undertaken this type of research in this region of Europe, namely, in Slovakia and the Czech Republic. The obtained results are the first to confirm the data included in the last EMCDDA and EUROPOL report, which indicates that the retail market for cocaine is expanding in Eastern Europe. Moreover, the conducted research is also the first to corroborate the data available from the last EMCDDA and EUROPOL report, which demonstrates a wide production scale of amphetamine in Poland. As part of the study, a comparative analysis of the concentration levels of the investigated drugs of abuse from this study and in sewage samples collected from different European regions as well as from East Asia and North America was performed.

Owing to the development and application of proper analytical protocols, it is possible to gain new information on the content of different groups of illegal drugs of abuse, originating from human excretion, in wastewater samples collected at a WWTP. Based on this information, we can monitor changing patterns of illicit drug consumption (designer drugs), transport processes and chemical, photochemical and biological transformations of hazardous drugs of abuse. The obtained results complement and expand upon the studies of drug use, enlarging the sewage-based epidemiology database. The proposed procedure can be used as a tool for tracking and estimating drug use in a population in real time, helping social scientists and authorities combat drug abuse. The obtained research results may also constitute a valuable database for managing the quality of the environment and preventing the introduction of these hazardous substances into the environment, thus reducing the negative impact on quality of life.

## Supplementary information

Figure Legends.

Fig.S1.

Fig.S2.

## References

[1] Chen C, Kostakis C, Irvine RJ, White JM (2013). Increases in use of novel synthetic simulant are not directly linked to decreased use of 3,4-methylenedioxy-N-methylamphetamine. Forensic Sci. Int..

[2] Thomas KV (2012). Comparing illicit drug use in 19 European cities through sewage analysis. Sci. Total. Environ..

[3] EMCDDA. European Drug Report 2018: Trends and Developments. (European Monitoring Centre for Drugs and Drug Addiction, Luxembourg, 2018).

[4] UNODC. Annual Report. Covering activities during 2017. (United Nations Office On Drugs and Crime, Viena, 2017).

[5] EMCDDA. European Drug Report. Trends and Developments. (European Monitoring Centre for Drugs and Drug Addiction, Luxembourg, 2016).

[6] UNODC. World Drug Report 2015. (United Nations Office On Drugs and Crime, New York, 2015).

[7] EMCDDA & Europol. EU Drug Markets Report 2019. (European Monitoring Centre for Drugs and Drug Addiction and Europol, Luxembourg, 2019).

[8] Yadav MK (2017). Occurrence of illicit drugs in water and wastewater and their removal during wastewater treatment. Water Res..

[9] Raktim P, Mallavarapu M, Kirkbride KP, Naidu R (2013). Illicit drugs and the environment — A review. Sci. Total. Environ..

[10] Boerman, F., Grapendaal, M., Nieuwenhuis, F. & Stoffers, E. 2017 national threat assessment: Organised crime. (National Police of the Netherlands, Driebergen, 2017).

[11] Reid MJ, Baz-Lomba JA, Ryu Y, Thomas KV (2014). Using biomarkers in wastewater to monitor community drug use: A conceptual approach for dealing with new psychoactive substances. Sci. Total. Environ..

[12] Nuijs ALNV (2011). Illicit drug consumption estimations derived from wastewater analysis: A critical review. Sci. Total. Environ..

[13] Nuijs, A. L. N. V. *et al*. Optimization, validation, and the application of liquid chromatography-tandem mass spectrometry for the analysis of new drugs of abuse in wastewater. Drug Testing and Analysis, Published online in Wiley Online Library (2013). 10.1002/dta.1460 23420682

[14] Castiglioni S, Thomas KV, Kasprzyk-Hordern B, Vandamd L, Griffiths P (2014). Testing wastewater to detect illicit drugs: State of the art, potential and research needs. Sci. Total. Environ..

[15] Lam LP (2015). Validation of the Drug Abuse Screening Test (DAST-10): A study on illicit drug use among Chinese pregnant women. Sci. Rep..

[16] Daglioglu N, Guzel E, Kilercioglu S (2019). Assessment of Illicit Drugs in Wastewater and Estimation of Drugs of Abuse in Adana Province, Turkey. Forensic Sci. Int..

[17] Foppe, K. S. & Subedi, B. Analysis of Illicit Drugs in Wastewater Using High-Performance Liquid Chromatography-Electrospray Ionization-Tandem Mass Spectrometry (HPLC-ESI-MS/MS). *Analysis of Drugs of Abuse*, 183–191, 10.1007/978-1-4939-8579-1_16 (2018). 10.1007/978-1-4939-8579-1_16 29974428

[18] Burgard DA, Banta-Green C, Field JA (2014). Working Upstream: How Far Can You Go with Sewage-Based Drug Epidemiology?. Ennvironmental Sci. Technol..

[19] Saleh A, Yamini Y, Faraji M, Rezaee M, Ghambarian M (2009). Ultrasound-assisted emulsification microextraction method based on applying low density organic solvents followed by gas chromatography analysis for the determination of polycyclic aromatic hydrocarbons in water samples. J. Chromatogr. A.

[20] Khan U (2014). Application of a sewage-based approach to assess the use of ten illicit drugs in four Chinese megacities. Sci. Total. Environ..

[21] Daughton, C. G. Vol. 791 (eds Daughton, C. G. & Jones-Lepp, T.) 348–364 (Americal Chemical Society, Washington, 2001).

[22] Zuccato E, Castiglioni S, Fanelli R (2005). Identification of the pharmaceuticals for human use contaminating the Italian aquatic environment. J. Hazard. Mater..

[23] Krizman I, Senta I, Ahel M, Terzic S (2016). Wastewater-based assessment of regional and temporal consumption patterns of illicit drugs and therapeutic opioids in Croatia. Sci. Total. Environ..

[24] Baker DR, Kasprzyk-Hordern B (2011). Critical evaluation of methodology commonly used in sample collection, storage and preparation for the analysis of pharmaceuticals and illicit drugs in surface water and wastewater by solid phase extraction and liquid chromatography–mass spectrometry. J. Chromatogr. A.

[25] Zuccato E, Castiglioni S, Bagnati S, Fanelli R (2008). Illicit drugs, a novel group of environmental contaminants. Water Res..

[26] Zuccato E, Chiabrando C, Castiglioni S, Bagnati R, Fanelli R (2008). Estimating Community Drug Abuse by Wastewater Analysis. Environ. Health Perspect..

[27] Castiglioni S (2006). Identification and Measurement of Illicit Drugs and Their Metabolites in Urban Wastewater by Liquid Chromatography-Tandem Mass Spectrometry. Anal. Chem..

[28] Berset JD, Brenneisen R, Mathieu C (2010). Analysis of llicit and illicit drugs in waste, surface and lake water samples using large volume direct injection high performance liquid chromatography – Electrospray tandem mass spectrometry (HPLC–MS/MS). Chemosphere.

[29] Bones J, Thomas KV, Paull B (2007). Using environmental analytical data to estimate levels of community consumption of illicit drugs and abused pharmaceuticals. J. Environ. Monit..

[30] Bijlsma L, Sancho JV, Pitarch E, Ibáñez M, Hernández F (2009). Simultaneous ultra-high-pressure liquid chromatography–tandem mass spectrometry determination of amphetamine and amphetamine-like stimulants, cocaine and its metabolites, and a cannabis metabolite in surface water and urban wastewater. J. Chromatogr. A.

[31] Pal R, Megharaj M, Kirkbride KP, Naidu R (2013). Illicit drugs and the environment — A review. Sci. Total. Environ..

[32] Klupczynska A, Dereziński P, Krysztofiak J, Kokot ZJ (2016). Estimation of drug abuse in 9 Polish cities by wastewater analy. Forensic Sci. Int..

[33] Reid MJ, Derrya L, Thomasa KV (2014). Analysis of new classes of recreational drugs in sewage: Synthetic cannabinoids and amphetamine-like substances. Drug. Test. Anal..

[34] Kasprzyk-Hordern B, Dinsdale RM, Guwy AJ (2009). Illicit drugs and pharmaceuticals in the environment – Forensic applications of environmental data. Part 1: Estimation of the usage of drugs in local communities. Environ. Pollut..

[35] Chiaia A, Banta-Green C, Field J (2008). Eliminating solid phase extraction with large-volume injection LC/MS/MS: analysis of illicit drugs and legal drugs and human urine indicators in US wastewaters. Environmenal Sci. Technol..

[36] Irvine RJ (2011). Population drug use in Australia: A wastewater analysis. Forensic Sci. Int..

[37] Lai FY (2013). Estimating daily and diurnal variations of illicit drug use in Hong Kong: A pilot study of using wastewater analysis in an Asian metropolitan city. Forensic Sci. Int..

[38] Foppe KS, Hammond-Weinberger DR, Subedi B (2018). Estimation of the consumption of illicit drugs during special events in two communities inWestern Kentucky, USA using sewage epidemiology. Sci. Total. Environ..

[39] Gao, J. *et al*. Could wastewater analysis be a useful tool for China? — A review. *Journal of Environmental Sciences*, 70–79 (2015). 10.1016/j.jes.2014.09.025 25597664

[40] Cosenza A (2018). Occurrence of illicit drugs in two wastewater treatment plants in the South of Italy. Chemosphere.

[41] Castiglioni S, Zuccato E, Chiabrando C, Fanelli R, Bagnati R (2008). Mass spectrometric analysis of illicit drugs in wastewater and surface water. Mass. Spectrometry Rev..

[42] Płotka J (2013). Green chromatography. J. Chromatogr. A.

[43] Vazquez-Roig P, Andreu V, Blasco C, Morillas F, Pico Y (2012). Spatial distribution of illicit drugs in surface waters of the natural park of Pego-Oliva Marsh (Valencia, Spain). Environ. Sci. Pollut. Res..

[44] Konieczka P, Wolska L, Namieśnik J (2010). Quality problems in determination of organic compounds in environmetal samples, such as PaHs and PCBs. Trends Anal. Chem..

[45] EC. In Council Directive 96/23/EC Vol. 221 (ed European Parliament) 1–29 (Official Journal of the European Communities, Brussels, 2002).

[46] Sulej-Suchomska AM (2016). Solid phase microextraction–comprehensive two-dimensional gas chromatography–time-of-flight mass spectrometry - A new tool for determination of polycyclic aromatic hydrocarbons in airport runoff water samples. Anal. Methods.

[47] REITOX. 2014 Naional Report to the EMCDDA by the Polish REITOX Focal Point. (National Bureau for Drug Prevention, Warsaw, 2014).

[48] Li K, Du P, Xu Z, Gao T, Li X (2016). Occurrence of illicit drugs in surface waters in China. Environmenal Pollut..

[49] Du P (2015). Methamphetamine and ketamine use in major Chinese cities, a nationwide reconnaissance through sewage-based epidemiology. Water Res..

[50] Kasprzyk-Hordern B, Dinsdale RM, Guwy AJ (2007). Multi-residue method for the determination of basic/neutral pharmaceuticals and illicit drugs in surface water by solid-phase extraction and ultra performance liquid chromatography–positive electrospray ionisation tandem mass spectrometry. J. Chromatogr. A.

[51] Baker D, Kasprzyk-Hordern B (2011). Multi-residue analysis of drugs of abuse in wastewater and surface water bysolid-phase extraction and liquid chromatography–positive electrosprayionisation tandem mass spectrometry. J. Chromatogr. A.

[52] González-Mariño I (2018). Multi-residue determination of psychoactive pharmaceuticals, illicit drugs and related metabolites in wastewater by ultra-high performance liquid chromatography-tandem mass spectrometry. J. Chromatogr. A.

[53] Senta, I., Krizman, I., Ahel, M. & Terzic, S. Multiresidual analysis of emerging amphetamine-like psychoactive substances in wastewater and river water. *Journal of Chromatography A* **1425** (2015). 10.1016/j.chroma.2015.11.043 26607313

[54] Mackuľak T (2016). Dominant psychoactive drugs in the Central European region: A wastewater study. Forensic Sci. Int..

[55] Baz-Lomba JA, Reid MJ, Thomas KV (2016). Target and suspect screening of psychoactive substances in sewagebased samples by UHPLC-QTOF. Analytica Chim. Acta.

[56] Vazquez-Roig P, Andreu V, Blasco C, Picó Y (2010). SPE and LC-MS/MS determination of 14 illicit drugs in surface waters from the Natural Park of L’Albufera (València, Spain). Anal. Bioanal. Chem..

[57] Celma A (2019). Simultaneous determination of new psychoactive substances andillicit drugs in sewage: Potential of micro-liquid chromatographytandem mass spectrometry in wastewater-based epidemiologyAlberto. J. Chromatogr. A.

[58] Nuijs ALNV (2014). Optimization, validation, and the applicationof liquid chromatography-tandem massspectrometry for the analysis of new drugsof abuse in wastewater. Drug. Anal..

[59] FDA. (eds United States Department of Health and Human Services & Food and Drug Administration) (Biopharmaceutics, Silver Spring, 2018).

[60] EMA. In EMEA/CHMP/EWP/192217/2009 (eds European Medicines Agency & Committee for Medicinal Products for Human Use) (European Medicines Agency, London, 2015).

[61] Ostman M, Fick J, Näsström E, Lindberg R (2014). A snapshot of illicit drug use in Sweden acquired through sewage water analysis. Sci. Total. Env..

[62] McCall A-K (2016). Critical review on the stability of illicit drugs in sewers and wastewater samples. Water Res..

[63] Castiglioni S (2006). Identification and measurement of illicit drugs and their metabolites in urban wastewater by liquid chromatography-tandem mass spectrometry. Anal. Chem..

[64] Castiglioni S, Borsotti A, Senta I, Zuccato E (2015). Wastewater analysis to monitor spatial and temporal patterns of use of two synthetic recreational drugs, ketamine and mephedrone, in Italy. Env. Sci. Technol..

[65] Causanilles A (2017). Improving wastewater-based epidemiology to estimate cannabis use: focus on the initial aspects of the analytical procedure. Analytica Chim. Acta.

[66] Chen C, Kostakis C, Irvine RJ, White JM (2013). Increases in use of novel synthetic stimulant are not directly linked to decreased use of 3,4-methylenedioxy-N-methylamphetamine (MDMA). Forensic Sci. Int..

[67] Ort C (2014). Spatial differences and temporal changes in illicit drug use in Europe quantified by wastewater analysis. Addiction.

[68] Yargeau V, Taylor B, Li H, Rodayan A, Metcalfe C (2014). Analysis of drugs of abuse in wastewater from two Canadian cities. Sci. Total. Env..

[69] Baker D, Kasprzyk-Hordern B (2013). Spatial and temporal occurrence of pharmaceuticals and illicit drugs in the aqueous environment and during wastewater treatment: new developments. Sci. Total. Env..

