# Supplementary material for: Urban wastewater analysis as an effective tool for monitoring illegal drugs, including new psychoactive substances, in the Eastern European region
Source: Sci Rep. 2020 Mar 17;10:4885. doi: 10.1038/s41598-020-61628-5 (PMC7078280; doi:10.1038/s41598-020-61628-5)
Supplement: Supplementary file 1 — Figure Legends. [file 41598_2020_61628_MOESM1_ESM.pdf]

Urban wastewater analysis as an effective tool for monitoring illegal drugs, including new psychoactive substances, in the Eastern European region.

Anna M. Sulej-Suchomska[a*], Agnieszka Klupczynska [b], Paweł Derezinski[b], Jan Matysiak[b], Piotr Przybyłowski[a], Zenon J. Kokot[b]

Figure Legends

Fig. 1 Schematic representation of the analytical procedure for the determination of illicit drugs in wastewater samples by using an SPE-HPLC-MS/MS-based system

Fig. 2 The concentrations of drugs of abuse in wastewater samples collected at a WWTP in Poznań (Poland) in autumn 2015

Fig. 3 The concentrations of illicit drugs in wastewater samples collected at a WWTP in Poznań (Poland) in spring 2016

Supplementary Information

Fig. S1 Extracted ion chromatograms of a standard solution of drugs of abuse acquired in MRM acquisition mode

Fig. S2 Chromatograms of wastewater samples collected at a WWTP in Poland on March 21, 2016 and analysed by using an SPE-HPLC-MS/MS-based procedure
