# Supplementary material for: Urban wastewater analysis as an effective tool for monitoring illegal drugs, including new psychoactive substances, in the Eastern European region
Source: Sci Rep. 2020 Mar 17;10:4885. doi: 10.1038/s41598-020-61628-5 (PMC7078280; doi:10.1038/s41598-020-61628-5)

Urban wastewater analysis as an effective tool for monitoring illegal drugs, including new psychoactive substances, in the Eastern European region.

Anna M. Sulej-Suchomska[a*], Agnieszka Klupczynska [b], Paweł Derezinski[b], Jan Matysiak[b], Piotr Przybyłowski[a], Zenon J. Kokot[b]

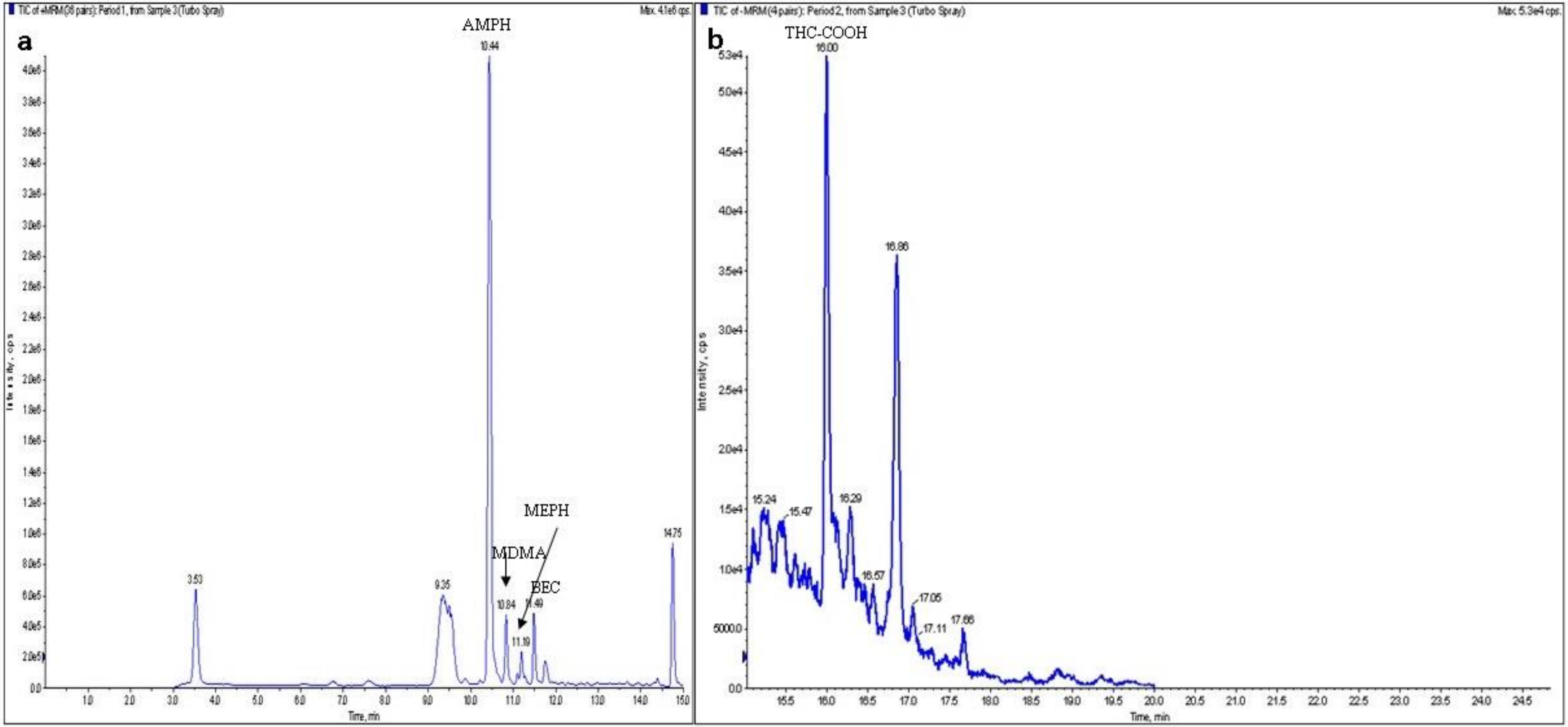

Supplement: Supplementary file 3 — Fig.S2. [file 41598_2020_61628_MOESM3_ESM.pdf]
